# Stacking of antithrombotic drugs in cardiology: evidence-based customisation

**DOI:** 10.1007/s12471-022-01699-3

**Published:** 2022-05-09

**Authors:** R. J. G. Peters

**Affiliations:** 1 Department of Cardiology, Amsterdam University Medical Centre, location VUmc, Amsterdam, The Netherlands; 2 Department of Cardiology, Amsterdam University Medical Centre, location AMC, Amsterdam, The Netherlands

The combination of an indication for chronic anticoagulant therapy, such as atrial fibrillation or a heart valve prosthesis, with an indication for a platelet inhibitor, usually the presence of coronary artery disease, requires very careful selection of a regimen for antithrombotic therapy. The bleeding complications that result from excessive treatment, particularly intracranial and gastrointestinal bleeding, are at least as harmful and potentially fatal as the thrombotic complications that occur with insufficient treatment, such as stent thrombosis and recurrent myocardial infarction. Pharmacological options include different numbers of drugs (‘dual therapy’, ‘triple therapy’), different drug strengths, different dosing and different durations of treatment. Paradoxically, with increasing numbers of publications on the subject, the questions and discussions have a tendency to multiply as opposed to being settled.

Adding to the complexity, direct oral anticoagulant drugs (DOACs) have largely replaced vitamin K antagonists (VKAs) in the treatment of atrial fibrillation, based on similar efficacy and a lower risk of bleeding. However, data on their use in combination with one or two platelet inhibitors, mostly in patients with coronary artery disease, and particularly those who are treated with percutaneous coronary interventions, are incomplete. Guidelines provide general advice, and clinicians seek additional support in reviews, position papers and consensus documents that summarise the results from randomised controlled trials and data from observational studies. Meanwhile, registry data show large variations in practice patterns across centres and countries. The article by de Veer et al. [1] on the WOEST 2 registry, published in this issue of the *Netherlands Heart Journal*, confirms these variations and tracks the trends over time in selecting specific anticoagulants (e.g. DOACs) and antiplatelet agents (e.g. clopidogrel). Registries such as WOEST 2 expand our understanding of practice patterns and identify the impact of new publications. Bleeding is again an important component of the outcomes in WOEST 2, with a concerning average incidence of 16%. The findings in 758 patients suggest that the results of dual therapy are comparable to those of triple therapy, and they provide support for the use of both DOACs and VKAs in these combinations.

However, there are limitations to the study that need to be considered. First, it carries the inherent limitations of an observational study, particularly selection bias and indication bias. Second, significant selection of cases into the registry is apparent from the fact that per centre (*n* = 9) an average of only 16 patients were included per year. Third, the observation time of 30 days does not completely capture the exposure of the patients to the benefits and downsides of antithrombotic treatment. Fourth, patients discharged on dual therapy were generally sicker than patients discharged on triple therapy. Fifth, patients on VKAs had different indications for anticoagulant therapy than patients discharged on DOACs.

The authors conclude that more research is needed before the best strategy can be selected. In addition to this quest for evidence and the need for more detailed guidelines, we will need to accept that a clear and uniform answer may not be possible. It may not even be desirable. Given the wide variation in patients, their ailments and concomitant medication, their coronary anatomy, the results of their individual percutaneous interventions, including stent type, and their personal circumstances, it is unlikely that a decision tree will ever outperform a prudent clinician who is aware of the available evidence. Best practice would therefore be a management decision that is tailored to the individual patient, based on the available literature, discussed with the patient and explained in the patient’s file. This is the very nature of medicine, and it is ‘evidence-based medicine’ (EBM) by definition, irrespective of whether the specific, tailored regimen can be found in a guideline, an online tool, a decision tree or a protocol. The importance of establishing that tailoring of drug regimens is in fact EBM lies in possible allegations of not ‘following guidelines’. Detrimental consequences of such views may include legal liability for physicians and reimbursement issues for patients.

The way out of these insecurities is to accept evidence-based customisation as a better approach than protocolised medicine.
